# Mimulone-Induced Autophagy through p53-Mediated AMPK/mTOR Pathway Increases Caspase-Mediated Apoptotic Cell Death in A549 Human Lung Cancer Cells

**DOI:** 10.1371/journal.pone.0114607

**Published:** 2014-12-09

**Authors:** Hyun-Kyu An, Kyoung-Sook Kim, Ji-Won Lee, Mi-Hyun Park, Hyung-In Moon, Shin-Ji Park, Ji-Sue Baik, Cheorl-Ho Kim, Young-Choon Lee

**Affiliations:** 1 College of Natural Resources and Life Science, Dong-A University, Busan, 604-714, South Korea; 2 Molecular and Cellular Glycobiology Unit, Department of Biological Sciences, SungKyunKwan University, Kyunggi-Do, 440-746, South Korea; Swedish Medical Center, United States of America

## Abstract

Anticancer properties and mechanisms of mimulone (MML), *C*-geranylflavonoid isolated from the *Paulownia tomentosa* fruits, were firstly elucidated in this study. MML prevented cell proliferation in a dose- and time-dependent way and triggered apoptosis through the extrinsic pathway in A549 human lung adenocarcinoma cells. Furthermore, MML-treated cells displayed autophagic features, such as the formation of autophagic vacuoles, a primary morphological feature of autophagy, and the accumulation of microtubule-associated protein 1 light chain 3 (LC3) puncta, another typical maker of autophagy, as determined by FITC-conjugated immunostaining and monodansylcadaverine (MDC) staining, respectively. The expression levels of LC3-I and LC3-II, specific markers of autophagy, were also augmented by MML treatment. Autophagy inhibition by 3-methyladenine (3-MA), pharmacological autophagy inhibitor, and shRNA knockdown of Beclin-1 reduced apoptotic cell death induced by MML. Autophagic flux was not significantly affected by MML treatment and lysosomal inhibitor, chloroquine (CQ) suppressed MML-induced autophagy and apoptosis. MML-induced autophagy was promoted by decreases in p53 and p-mTOR levels and increase of p-AMPK. Moreover, inhibition of p53 transactivation by pifithrin-α (PFT-α) and knockdown of p53 enhanced induction of autophagy and finally promoted apoptotic cell death. Overall, the results demonstrate that autophagy contributes to the cytotoxicity of MML in cancer cells harboring wild-type p53. This study strongly suggests that MML is a potential candidate for an anticancer agent targeting both autophagy and apoptotic cell death in human lung cancer. Moreover, co-treatment of MML and p53 inhibitor would be more effective in human lung cancer therapy.

## Introduction

Lung cancer is the most prevalent malignant tumor that represents one of the leading causes of global cancer-associated death and non-small cell lung carcinoma (NSCLC) captures almost 85% of all lung cancers [1], [2]. Despite considerable advances in lung cancer therapy including surgery, radiotherapy and chemotherapy, the prognosis for patients having lung cancer is still poor, with less than 15% of overall 5-year survival rate [1]. Especially, chemotherapy using platinum compounds or platinum-based combinations is the most frequently used lung cancer therapy and is considered to be the optimal treatment in patients having advanced-stage NSCLC [2], [3]. However, the efficacy of chemotherapy in patients with advanced lung cancer is extremely limited, due to drug resistance and toxic side effects of drugs [2], [3]. Thus, it is crucial to develop less toxic and more effective chemotherapeutic agents for treating advanced lung cancer patients.

In recent years, plant-derived natural products have received extensive attention as main sources of new drugs for reducing chemotherapy-associated side effects and they exert their anticancer effects by triggering apoptosis and autophagy [4]–[7]. Recent studies have demonstrated that several plant-derived natural products, including plumbagin [8], glossogin [9], curcumin [10], celastrol [11], isolinderalactone [12], glycyrrhizin [13], polydatin [14], 6-shogaol [15], glycyrrhetinic acid [16] and embelin [17], induce apoptosis through the intrinsic and/or extrinsic pathway and activation of p38/JNK pathway in human lung cancer cells. In addition, 6-shogaol caused cell death through autophagy induction by the inhibition of the AKT/mTOR pathway in human NSCLC A549 cells [18] and paclitaxel and feroniellin A exerted their cytotoxic effects by inducing both autophagy and apoptosis in human lung cancer A549 cells [19], [20].

*Paulownia tomentosa* Steud. (Scrophulariaceae) is deciduous tree distributed throughout China, Korea, and Japan [21] and extracts from *P*. *tomentosa* have been used to relieve bronchitis, asthmatic attacks and phlegm in traditional Chinese medicine [22]. Previous studies demonstrated that *C*-geranylated flavonoids, the main bioactive constituents of *P. tomentosa*, have neuroprotective effects, antiradical and antibacterial activities [23]-[26]. In addition, *C*-geranylated flavanones from *Paulownia tomentosa* fruits exhibited strong cytotoxic activity in various human cancer cell lines [27], [28]. It has also been recently reported that geranylated flavanone tomentodiplacone B directly inhibits cell proliferation by down-regulation of cyclin-dependent kinase 2 activity, leading to G1 phase accumulation in THP-1 human monocytic leukaemia cells [29]. However, the underlying mechanism responsible for antitumor activity of geranylated flavonoids is not well elucidated.

We have recently isolated a compound belonging to *C*-geranylated flavonoids, mimulone (MML), from the *Paulownia tomentosa* fruits. In the present study, we firstly examined the anticancer effects of MML on human lung cancer cells and also clarified its mechanism of action. We demonstrate here that MML triggers autophagy preceding apoptosis in human NSCLC A549 cells, and autophagy inhibition decreases apoptosis in MML-treated cells.

## Materials and Methods

### Materials

**Figure 1:**
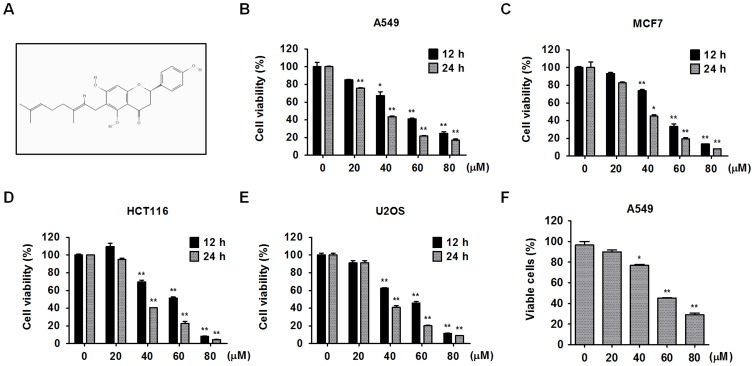
The molecular structure of MML and its cytotoxic effects on various cancer cell lines. (A) Molecular structure of the mimulone. Various human cancer cells, non-small cell lung cancer A549 (B), breast cancer MCF7 (C), colon cancer HCT116 (D) and osteosarcoma U2OS (E) cells were treated with the MML as indicated concentrations (0-80 µM) for 12 h or 24 h and then cell viability was measured by MTT assay. Bar graphs indicate the percentage of viability. (F) A549 cells were treated with the MML at various concentrations (0-80 µM) for 24 h and viable cells were counted by trypan blue staining. Live cells (non-stained cells) were calculated using hemocytometer and bar graph indicates the percentage of viable cells. All data were expressed as mean ± SEM of three independent experiments. *p<0.05 and **p<0.01 compared with control.

Monodansylcadaverine (MDC), 4′, 3-methyladenine (3-MA), chloroquine (CQ), compound C (comp C) and 6-diamidino-2-phenylindole dihydrochloride (DAPI) were purchased from Sigma-Aldrich (St. Louis, MO, USA). Z-VAD-FMK (pan-caspase inhibitor), Z-DEVD-FMK (caspase-3 inhibitor), Z-IETD-FMK (caspase-8 inhibitor) and Z-LEHD-FMK (caspase-9 inhibitor) were obtained from Calbiochem (EMD Biosciences, San Diego, CA, USA). 3-(4, 5-Dimethylthiazol-2-yl)-2, 5-diphenyltetrazolium bromide (MTT), DAPI, puromycin-dihydrochloride and chloroquine (CQ) were dissolved in dH_2_O. 3-MA (100 mM), poly-L-Lysine (0.1%) and polybrene (20 mg/ml) dissolved in phosphate buffered saline (PBS). Pifithrin-α (PFT-α), compound C (comp C) and MDC were dissolved in dimethyl sulfoxide (DMSO). Antibodies for ATG7, Beclin-1, LC3, caspase-3, caspase-8, caspase-9, Bid, p-p38, p38, p-AMPK-α, AMPK-α, p-ACC, ACC, p-mTOR, mTOR were obtained from Cell Signaling Technology (Dancers, Mass, USA); antibodies for PARP-1/2, p53 (DO-1), p-ERK and ERK were purchased from Santa Cruz Biotechnology (Santa Cruz, CA, USA); antibody for p62/SQSTM1 was purchased from Sigma-Aldrich (St. Louis, MO, USA); antibody for glyceraldehyde-3-phosphate dehydrogenase (GAPDH) was obtained from Millipore (Milford, MA, USA); Horseradish peroxidase (HRP)-conjugated secondary antibodies were purchased from Cell signaling Technology (Dancers, Mass, USA) and Enzo Life Science (Farmingdale, NY, USA), respectively; Fluorescein isothiocyanate (FITC)-conjugated secondary antibody was obtained from Vector Laboratories (Burlingame, CA, USA). Annexin V-FITC apoptosis detection kit and BCA protein assay kit were purchased from BD Biosciences (San Jose, CA, USA) and Thermo (Rockford, IL, USA), respectively. MML (Fig. 1A) isolated from *P. tomentosa* fruits was prepared as described in previous report [25], dissolved in DMSO at 40 mM for stock concentration, and stored at -20°C.

### Cell cultures

Human non-small cell lung carcinoma A549, human breast adenocarcinoma MCF-7, human colorectal carcinoma HCT116 and human osteosarcoma U2OS cell lines were purchased from American Type Culture Collection (Rockville, MD, USA). Cells were maintained in Dulbecco's modified Eagle's medium (DMEM; WelGENE Co., Daegu, Korea) containing 10% (v/v) heat-inactivated fetal bovine serum (FBS) and 1% PSA (WelGENE Co., Daegu, Korea) at 37°C in a humidified atmosphere of 5% CO_2_ in air.

### Cell viability

For analysis of cell viability, MTT assay was carried out by a procedure similar to that described previously [30]. Briefly, cells were seeded in 24-well plate (5×10^4^ cells/well) and grown for 24 h. The cells were then treated with MML at different concentrations (0-80 µM) for 24 h or treated with 60 µM of MML for different time courses (0-24 h). After MML treatment, cells were incubated with medium/MTT (0.5 mg/ml) mixture for 3 h. DMSO was added to dissolve the reduced formazan crystal from MTT and absorbance was read at 540 nm using ELISA plate reader (Bio-Rad, Hercules, CA, USA). To confirm the effect of MML on cell proliferation, live cells were counted by trypan blue staining according to the manufacturer's instructions. In brief, A549 cells were plated in 12-well plate and treated with MML as indicated concentrations for 24 h. After treatment, cells were harvested using trypsin-EDTA (WelGENE Co., Daegu, Korea) and stained with 0.4% trypan blue solution (Gibco, Carlsbad, CA). Living cells were counted by hemocytometer. Cell viability was shown relative to the control.

### Protein extraction and western blot analysis

Whole-cell protein extracts from A549 cells were prepared with a RIPA buffer (Pierce, Rockford, IL, USA) containing protease inhibitor mix (GE Healthcare, Piscataway, USA) and phosphatase inhibitor cocktail (Thermo, Rockford, IL, USA). Proteins from whole-cell lysates were quantified using BCA protein assay kit according to the manufacturer's instructions. Western blot analysis was carried out as described previously [30]. Enhanced chemiluminescence (ECL) detection system (Amersham Bioscience, NJ, USA) was used to detect signal and signal intensity of detected protein bands were measured using Scion Image Instrument (Scion Co., Frederick, MD, USA). Equal loading was assessed by GAPDH as internal controls for Western blotting.

### Immunocytochemistry

Immunofluorescence staining was carried out by a similar procedure as previously described [30]. In brief, A549 cells were cultured on sterile coverslips and treated with MML for 24 h, and then fixed with 3% paraformaldehyde (PFA) for 15 min at 37°C. Next, permeabilization step was carried out with chilled methanol (100%) for 10 min at −20°C and then cells were subsequently incubated in a blocking solution containing 5% bovine serum albumin (BSA) and 1% Triton-X 100 for 1 h at 37°C. Cells were then incubated with the LC3 antibody for 12 h at 4°C followed by FITC-conjugated secondary antibody for 1 h at 37°C. Nuclei were stained with DAPI (1 µg/ml) for 10 min at 37°C. Fluorescence images were captured by LSM 700 confocal laser scanning microscope (Carl Zeiss, Oberkochen, Germany).

### MDC staining

MDC staining was also carried out by a procedure similar to that described previously [30]. Cells were fixed with 3% PFA for 10 min at 37°C, and then incubated with 50 µM monodansylcadaverine (MDC), an autofluorescent compound that labels autophagic vacuoles at 37°C for 10 min. Fluorescent images were captured by Olympus BX51 fluorescence microscope (Center Valley, PA, USA).

### Annexin V and PI staining

Cells were treated with as indicated concentrations of MML and/or inhibitors (caspase inhibitors, 3-MA, PFT-α) for 24 h, and harvested using trypsin-EDTA. After staining with FITC-conjugated Annexin V and propidium iodide (PI), apoptotic cells were measured using Beckman-Coulter Cytomics FC500 flow cytometer (Beckman-Coulter, Miami, FL, USA).

### RNA interference

pLKO.1 lentiviral expression plasmid containing shRNA against Beclin-1 (TRCN0000033552) and p53 (TRCN0000003753), (Beclin-1;5′-CCGGCTCAAGTTCATGCTGACGAATCTCGAGATTCGTCAGCATGAACTTGAGTTTTTG-3′, p53;5′-CCGGCGGCGCACAGAGGAAGAGAATCTCGAGATTCTCTTCCTCTGTGCGCCGTTTTT-3′) were purchased from Sigma-Aldrich (Mission shRNA). Viral particles were generated in 294T according to the manufacturer's instructions. In brief, cells were transfected with pLKO.1 shBeclin-1, VSVG and Gag vectors. Viral soup was then injected to A549 cell and incubated for 12 h. Virus-infected A549 cells were selected by Puromycin-dihydrochloride treatment and selected cells were used for experiments. pLKO.1 shRNA control vector (Mission shRNA, SHC002) is used as control.

### Statistical analysis

All data are expressed as means ± SEM of three independent experiments in each group. The differences between each groups were evaluated with Student's *t*-test and *p<0.05 was considered to show statistical significance.

## Results

### Effect of MML on cell viability of various human cancer cell lines

To assess the effects of MML on cell growth of various human cancer cell lines, human lung cancer A549 (Fig. 1B), human breast cancer MCF-7 (Fig. 1C), human colon cancer HCT116 (Fig. 1D) and human osteosarcoma U2OS (Fig. 1E) cells were treated with MML at different concentrations for 12 h or 24 h and then followed by MTT assays. MTT assay results showed that MML treatment significantly inhibited cell proliferation in a dose- and time-dependent way in these cancer cell lines. To further check the effect of MML on cell proliferation of A549 cells, cells were treated with MML at different concentrations for 24 h and then subjected to trypan blue staining. The result revealed a significant inhibition of cell proliferation by MML treatment in a dose-dependent way (Fig. 1F).

### MML triggers caspase-dependent apoptosis in A549 cells

**Figure 2:**
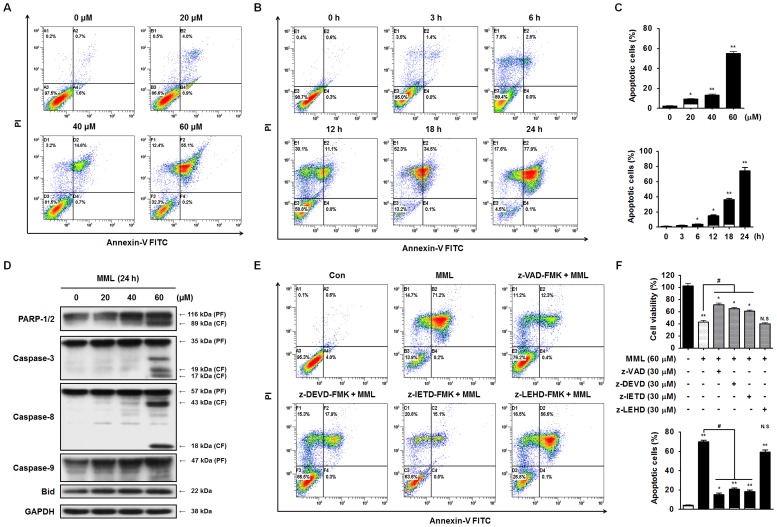
MML induces caspase-mediated apoptosis in A549 cells. (A) A549 cells were treated with the MML as indicated concentrations (0–60 µM) for 24 h and then stained with FITC- conjugated Annexin V and PI. Apoptotic cells were measured by flow cytometer. (B) A549 cells were treated with 60 µM of MML for a time dependent manner. Apoptotic cells were then stained with Annexin V and PI and detected using flow cytometer. (C) Bar graph indicates the percentage of apoptotic cells (dose-dependent manner; top, time-dependent manner; bottom). Apoptotic population was evaluated by Annexin V positive, PI negative (AV+/PI-, white bar) and Annexin V and PI double positive (AV+/PI+, black bar) apoptotic cells. (D) Cells were treated with the MML as indicated concentrations (0–60 µM) for 24 h and Western blot analysis was performed to check apoptosis marker, caspase−3, −8, −9, Bid and PARP-1/2, respectively. GAPDH was used as loading control of Western blot analysis. (E) Cells were pretreated with or without each caspase inhibitor, Z-VAD-FMK (30 µM), Z-DEVD-FMK (30 µM), Z-IETD-FMK (30 µM) and Z-LEHD-FMK (30 µM) for 1 h and then treated with MML (60 µM) for 24 h. Apoptotic cells were stained with Annexin V and PI and then populations were detected by flow cytometer. (F) Cells were treated with caspase inhibitors and MML under the same conditions as the flow cytometric analysis and then cell viability was measured by MTT assay. Bar graphs indicate the percentage of cell viability (top) and the percentage of apoptotic cells (bottom), respectively. Apoptotic population was evaluated by Annexin V positive, PI negative (AV+/PI-, white bar) and Annexin V and PI double positive (AV+/PI+, black bar) apoptotic cells. All data were expressed as mean ± SEM of three independent experiments. *p<0.05 and **p<0.01 compared with control; ^#^ p<0.05 compared with MML-treated group.

To determine whether MML-induced cytotoxicity in A549 cells was due to apoptosis, after MML treatment at different concentrations for 24 h or 60 µM of MML treatment for different time periods, apoptotic population was analyzed by Annexin V/PI double staining. Annexin V/PI-positive cells were markedly increased in a dose- and time-dependent way (Fig. 2A–C) indicating the apoptotic effect of MML on A549 cells. Flow cytometric analysis also revealed that MML treatment increased accumulation of cells at the apoptotic sub-G1 phase in a dose-dependent manner. The number of cells at G2/M phase also increased in a dose-dependent manner (S1 Figure). To further confirm the effect of MML on the induction of apoptosis in A549 cells, caspase-3 activation and poly (ADP-ribose) polymerase (PARP) cleavage were investigated by immunoblotting with their antibodies. Caspase-3, a key executioner in the apoptotic machinery, cleaves many proteins indispensable for cell survival and is activated through cleavage into two fragments (17 kDa and 19 kDa) by caspase-8 and/or caspase-9 during apoptosis [31], [32]. Activated caspase-3 subsequently cleaves cellular proteins, including PARP, which leads to apoptotic cell death. PARP plays a vital role in maintaining genomic integrity and is the major protein to be proteolyzed by caspase-3 during apoptosis [32]. As shown in Fig. 2D, caspase-3 and PARP cleavages were distinctly increased after 60 µM of MML treatment for 24 h, indicating that MML induced apoptosis through caspase-3 activation and PARP cleavage in A549 cells. Caspase-8 and caspase-9 play crucial roles in induction of apoptosis through the death receptor-mediated (extrinsic) and the mitochondrial (intrinsic) pathways, respectively [31], [32]. The cleavage of Bid is required for crosstalk between the extrinsic and intrinsic pathways [31], [32]. To unravel the pathway by which MML induces apoptosis in A549 cells, protein contents of caspase-8 and caspase-9 were analyzed by immunoblot analysis. As shown in Fig. 2D, MML treatment increased the cleaved forms (18 kDa and 43 kDa) of caspase-8, but it did not trigger caspase-9 and Bid cleavages, indicating that MML induced the activation of caspase-8 in A549 cells. To further confirm whether the MML-induced apoptosis was caspase-dependent, the percentage of apoptotic cells was checked by Annexin V-PI double staining after a 24-h treatment with various caspase inhibitors such as Z-VAD-FMK (pan-caspase inhibitor), Z-DEVD-FMK (caspase-3 inhibitor), Z-IETD-FMK (caspase-8 inhibitor) and Z-LEHD-FMK (caspase-9 inhibitor). As shown in Fig. 2E, flow cytometric analysis revealed that MML-induced apoptosis was rescued by pan-caspase inhibitor, caspase-3 and caspase-8 inhibitors when compared with MML treatment alone, but not by caspase-9 inhibitor. A similar result was also obtained from MTT assay (Fig. 2F). Taken together, these results suggest that MML-induced apoptosis is driven primarily by extrinsic pathway rather than intrinsic pathway.

### MML triggers autophagy in A549 cells

**Figure 3:**
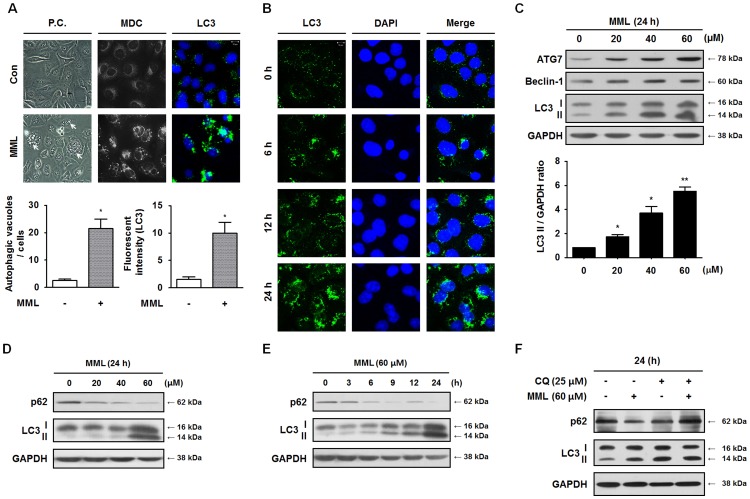
MML treatment induces autophagy in A549 cells. (A) A549 cells were treated with 60 µM of MML for 24 h and then morphological images were captured by phase contrast microscope (400×, P.C.). Arrows indicate the endogenous autophagosome-like vacuoles (left) and bar graph indicates the number of vacuoles per cell. Cells were treated with MML (60 µM, 24 h) and then cells were stained with MDC and LC3 antibody, respectively. MDC (bright color, middle) images were captured by fluorescence microscope and LC3 (green, right) fluorescent images were detected by confocal microscope (bar; 10 µm). Bar graph indicates the fluorescent intensity of LC3 FITC. (B) Cells were treated with MML (60 µM) for different time periods (0–24 h) and LC3 immunofluorescence staining was performed to detect autophagosomes. Nuclei were stained with DAPI. Images were captured using confocal microscope (bar; 10 µm). (C) A549 cells were treated with various concentrations of MML (0–60 µM) for 24 h. Western blot analysis was then performed with antibodies against ATG7, Beclin-1 and LC3, respectively. GAPDH was used as loading control. Bar graph indicates densitometry analysis of LC3-II/GAPDH ratio. A549 cells were treated with different concentrations of MML (0–60 µM) for 24 h (D) or 60 µM of MML for the different time course (E) and then immunoblot analysis was carried out using antibodies against p62 and LC3, respectively. GAPDH was used as loading control. (F) A549 cells were pre-incubated with or without chloroquine (CQ, 25 µM) for 1 h, then treated with MML (60 µM) for 24 h. Western blot analysis was performed using antibodies as indicated above. GAPDH was used as loading control of Western blotting. All data were expressed as mean ± SEM of three independent experiments. *p<0.05 and **p<0.01 compared with control.

Plant-derived natural compounds are known to exert their anticancer effects by inducing autophagy in human cancer cells [7]. Autophagy is a catabolic process forming double-membrane vesicles, termed autophagosomes [33]. To investigate whether MML triggers autophagy in A549 cells, the morphological changes after MML treatment were checked. As shown in Fig. 3A, extensive cytoplasm vacuolization and formations of autophagosome-like vacuoles in MML-treated A549 cells were observed under the phase contrast microscopy. To confirm autophagy induction by MML, MDC staining and microtubule-associated protein 1 light chain 3 (LC3) immunostaining using fluorescent antibodies to LC3 were performed. MDC and LC3 are known as specific markers of autophagic vacuoles and autophagosomes, respectively, and puncta formation of LC3 into the autophagosome can be observed by confocal microscopy, which is used widely as reliable method capable of detecting autophagy [33]. As shown in Fig. 3A, MML-untreated control cells showed diffused MDC-staining, whereas MML-treated A549 cells resulted in an increase of fluorescence intensity indicating extensive MDC-positive autophagic vacuoles. Moreover, the increased subcellular localization of punctate LC3 was also detected in MML-treated A549 cells compared with untreated control cells and LC3 puncta formation was increased in a time-dependent way (Fig. 3B). To further ascertain the autophagosome formation in MML-treated cells, the changes in the three major autophagy-related markers, LC3-II, Beclin-1 and ATG7, were analyzed by immunoblotting. ATG7 and LC3-II protein levels were remarkably increased in a dose-dependent way by MML treatment, but Beclin-1 level was slightly augmented (Fig. 3C). Together, these data definitely show that MML induces autophagosome formation in A549 cells.

It is known that autophagosomes also accumulate when autophagic flux indicating the whole process of autophagy is inefficient [35], [46]. To evaluate whether MML affects the autophagic flux in A549 cells, the level of p62 protein which is used to monitor autophagic flux [35], [46] was checked by immunoblotting with its antibody. As shown in Fig. 3D and 3E, the p62 level was markedly decreased by MML treatment in a dose- and time-dependent way. Moreover, to assess the effects of MML on autophagic vesicle turnover, the protein levels of p62 and LC3 II were checked by immunoblotting after a 24-h treatment with chloroquine (CQ) known as lysosomal degradation inhibitor. As shown in Fig. 3F, pretreatment with CQ significantly increased the levels of p62 and LC3 II compared to MML treatment alone. These results clearly suggest that MML treatment did not interfere with autophagic vesicle turnover. Collectively, these results certainly indicate that MML induced autophagy without impairment of autophagic flux in A549 cells.

### Inhibition of autophagy by autophagy inhibitor or knockdown of Beclin-1 suppresses MML-mediated apoptotic cell death

**Figure 4:**
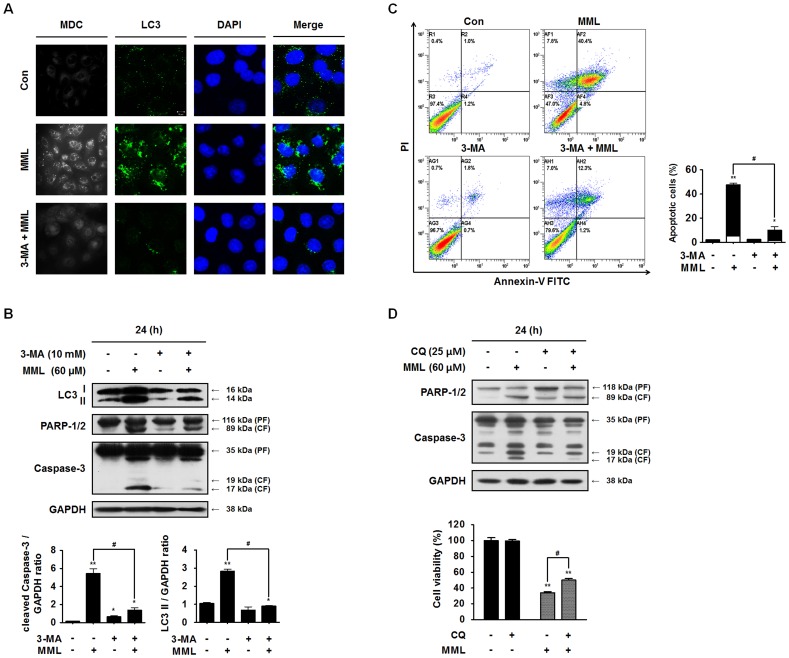
Inhibition of autophagy by 3-MA suppresses MML-induced apoptotic cell death. (A) A549 cells were pre-incubated with or without 3-MA (10 mM) for 3 h and then incubated with MML (60 µM) for 24 h. Cells were stained with MDC (bright color) or LC3 (green) antibody, respectively. Nuclei were stained with DAPI (blue). Fluorescent images were obtained using confocal microscope (bar; 10 µm). (B) Cells were pretreated with or without 3-MA for 3 h before treatment of MML (60 µM) for 24 h. Western blot analysis was performed with antibodies against LC3, PARP-1/2 and caspase-3, respectively. GAPDH was used as loading control. Bar graphs indicate the ratio of cleaved caspase-3/GAPDH and LC3-II/GAPDH, respectively. (C) A549 cells were pretreated 3 h with or without 3-MA and treated with MML (60 µM) for 24 h and cells were stained with FITC-conjugated Annexin V/PI and then measured by FACS. Bar graph indicates the percentage of apoptotic cells. Percentage of apoptotic cells was evaluated by Annexin V positive and PI negative (AV+/PI-, white bar) and Annexin V and PI double positive (AV+/PI+, black bar) apoptotic cells. (D) Cells were pretreated with or without CQ for 1 h and then incubated with MML for 24 h. Immunoblotting analysis was performed using antibodies as indicated before. Cells were treated with CQ and MML under the same conditions as mentioned before and then cell viability was evaluated by MTT assay (bottom). Bar graph indicates the percentage of cell viability. All data were expressed as mean ± SEM of three independent experiments. *p<0.05 and **p<0.01 compared with control; ^#^ p<0.05 compared with MML-treated group.

It has been recently documented that autophagy inhibition by a specific autophagy inhibitor, such as 3-methyladenine (3-MA), can promote apoptosis in human cancer cells [35]–[38]. Thus, we investigated the effect of 3-MA on the formation of LC3 puncta and autophagic vacuoles in MML-treated A549 cells. The punctate LC3 distribution and formation of MDC-positive autophagosomes in MML-induced cells were noticeably suppressed by 3-MA treatment (Fig. 4A). In addition, the co-treatment with MML and 3-MA significantly inhibited not only LC3-II accumulation but also caspase-3 and PARP cleavages (Fig. 4B). Then we checked the effect of 3-MA on the apoptosis induction using Annexin V/PI double staining. Annexin V/PI-positive cells were markedly decreased in cells co-treated with MML and 3-MA, compared with those treated with MML alone (Fig. 4C). Similar results were obtained by co-treatment of MML with CQ (Fig. 4D). These results obviously reveal that autophagy inhibition by 3-MA and CQ could suppress MML-induced apoptosis in A549 cells.

**Figure 5:**
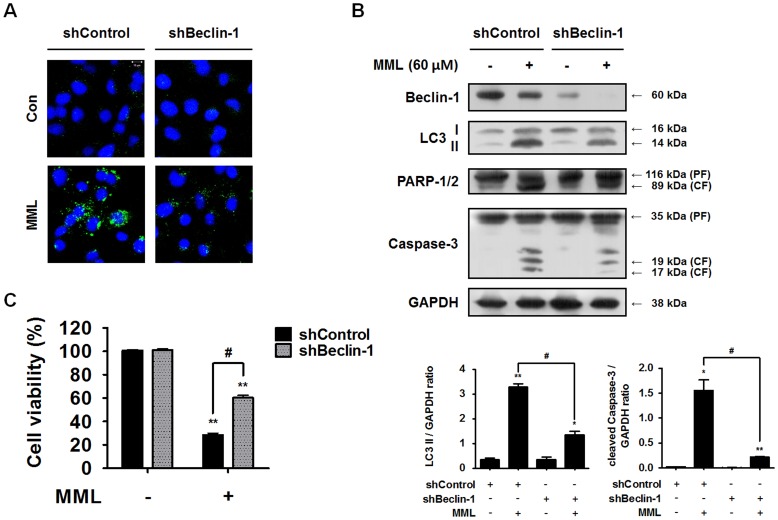
Knockdown of *Beclin-1* gene suppresses MML-induced apoptotic cell death. (A) Control and Beclin-1 knockdown cells were treated with MML (60 µM) for 24 h and then LC3 immunofluorescence staining was carried out for detecting autophagosomes. Nuclei were stained with DAPI. Images were captured by confocal microscope (bar; 10 µm). (B) Knockdown cells (shControl, shBeclin-1) were treated with MML (60 µM) for 24 h. Western blot analysis was performed with antibodies against Beclin-1, LC3, PARP-1/2 and caspase-3, respectively. GAPDH was used as loading control. Bar graphs indicate the ratio of LC3-II/GAPDH and cleaved caspase-3/GAPDH, respectively. (C) Control and Beclin-1 knockdown cells were treated with MML (60 µM) for 24 h and then cell viability was measured by MTT assay. Bar graph represents the percentage of cell viability. All data were expressed as mean ± SEM of three independent experiments. *p<0.05 and **p<0.01 compared with control; ^#^ p<0.05 compared with MML-treated group.

More specific inhibition of the autophagy pathway can be achieved by knockout or knockdown of autophagy-related (*ATG*) genes and *Beclin-1* gene. To further confirm whether autophagy can regulate apoptosis, Beclin-1, a critical regulator of autophagy, was knocked down using small hairpin RNA (shRNA) and then changes of endogenous autophagosomes were checked by LC3 immunostaining. As shown in Fig. 5A, endogenous LC3 distribution is markedly decreased in MML-treated Beclin-1 knockdown cells (shBeclin-1) compared with MML-treated shControl cells. Moreover, as shown in Fig. 5B, knockdown of *Beclin-1* gene markedly suppressed Beclin-1 protein expression and the accumulation of LC3-II compared to shControl. Consistent with the results using 3-MA and CQ, caspase-3 and PARP cleavages were also significantly inhibited by repression of autophagy through knockdown of *Beclin-1* gene. MTT assay result also showed that the cytotoxic effect of MML was significantly reduced by knockdown of *Beclin-1* gene (Fig. 5C). Taken together, these results clearly demonstrate that autophagy inhibition by 3-MA and CQ or Beclin-1 knockdown prevented MML-induced apoptosis in A549 cells.

### MML induces autophagy through decrease in p53 levels

**Figure 6:**
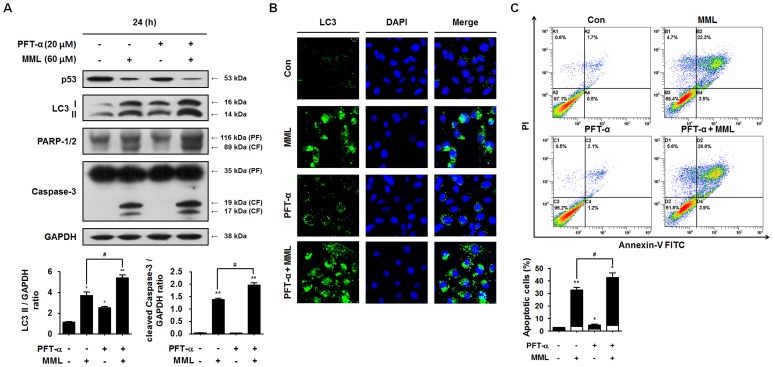
MML-induced autophagy is associated with the decrease in p53 levels. (A) A549 cells were co-treated with or without pifithrin-α (PFT-α, 20 µM) and MML (60 µM) for 24 h. After that, immunoblot analysis was performed with antibodies against p53, LC3, PARP-1/2 and caspase-3, respectively. Bar graphs indicate the ratio of LC3-II/GAPDH and the cleaved caspase-3/GAPDH, respectively. (B) A549 cells were co-treated with or without PFT-α (20 µM) and MML (60 µM) for 24 h and then immunofluorescence staining was performed using LC3 (green) antibody. Nuclei were stained with DAPI (blue). Fluorescent images were obtained using confocal microscope (Bar; 10 µm). (C) Cells were co-treated with or without PFT-α (20 µM) and MML (60 µM) for 24 h and apoptotic cells were stained with FITC-conjugated Annexin V/PI and analyzed by flow cytometry. Bar graph indicates the percentage of apoptotic cells. Percentage of apoptotic cells was evaluated by Annexin V positive and PI negative (AV+/PI-, white) and Annexin V and PI double positive (AV+/PI+, black) apoptotic cells. All data were expressed as mean ± SEM of three independent experiments. *p<0.05 and **p<0.01 compared with control; ^#^ p<0.05 compared with MML-treated group.

It has been recently reported that p53 plays a crucial role in the regulation of autophagy [39]–[41]. To investigate whether p53 is involved in MML-mediated autophagy, thus, the levels of p53 and LC3-II protein were examined by immunoblotting. As shown in Fig. 6A, MML treatment remarkably reduced p53 levels compared with untreated control cells, but increased LC3-II levels. In addition, caspase-3 and PARP cleavages were also increased by MML treatment. Furthermore, LC3-II levels and cleaved caspase-3 and PARP levels were significantly increased in cells co-treated with MML and pifithrin-α (PFT-α), a pharmacological p53 inhibitor, compared with those treated with MML or PFT-α alone. These results clearly indicate that reduction in p53 levels by MML could enhance induction of autophagy and apoptosis in A549 cells harboring wild-type p53. To further confirm the role of p53 in MML-induced autophagy, autophagosomes were investigated by LC3 immunostaining. As shown in Fig. 6B, a significant accumulation of LC3 puncta was observed in cells co-treated with MML and PFT-α, indicating augment of autophagy induction by p53 loss. In addition, the percentage of Annexin V/PI-positive cells was about 10% higher in cells co-treated with MML and PFT-α than those treated with MML alone (Fig. 6C). These results strongly suggest that MML-induced autophagy and apoptosis are closely related with p53 loss.

### MML induces autophagy through p53-mediated AMPK/mTOR pathway

**Figure 7:**
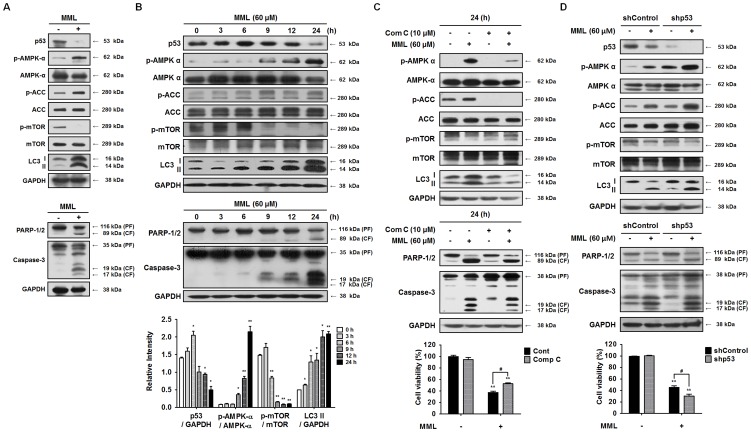
MML induces autophagy through p53-mediated AMPK/mTOR pathway. (A) A549 cells were treated with or without MML (60 µM) for 24 h and Western blot analysis was performed with antibodies as indicated above (p53, p-AMPK-α, AMPK-α, p-ACC, ACC, p-mTOR, mTOR, LC3, PARP-1/2, caspase-3). (B) A549 cells were treated with MML (60 µM) for the different time period as indicated above. Immunoblot analysis was performed with antibodies as indicated before. Bar graphs show the densitometry analysis of p53, p-AMPK and mTOR (bottom). Each graph indicates the ratios of p53/GAPDH, p-AMPK/AMPK, p-mTOR/mTOR and LC3 II/GAPDH, respectively. (C) A549 cells were treated with or without compound C (10 µM) for 1 h and treated with MML (60 µM) for 24 h. Immunoblotting analysis was performed with antibodies as indicated above. Cells were treated with same conditions as indicated before and then cell viability was measured by MTT assay. Bar graph represents the percent of cell viability (bottom). (D) Control cells and shp53 knockdown cells were treated with MML (60 µM) for 24 h and then Western blot analysis was performed with antibodies as indicated before. Cell viability was measured by MTT assay under the same conditions as mentioned above. Bar graph indicates the percent of cell viability (bottom). All data were expressed as mean ± SEM of three independent experiments. **p<0.01 compared with control; ^#^ p<0.05 compared with MML-treated group.

It is known that autophagy triggered by p53 loss is closely associated with AMP-activated protein kinase (AMPK) activation and an autophagy suppressor, the mammalian target of rapamycin (mTOR) inhibition [39]–[43]. To confirm whether p53-mediated AMPK/mTOR pathway is involved in MML-induced autophagy, therefore, the levels of p53 and the phosphorylated forms of AMPK and mTOR were examined by immunoblotting. As shown in Fig. 7A, the levels of p53 and phospho-mTOR were remarkably decreased by MML treatment, whereas the levels of phospho-AMPK and phospho-Acetyl-CoA Carboxylase (ACC), an AMPK substrate, were significantly increased. In addition, MML treatment also augmented LC3-II protein level as well as cleaved PARP and caspase-3 levels, compared with those of control cells. These results suggest that the levels of p53 reduced by MML treatment are correlated with the levels of activated AMPK and attenuated mTOR, which leads to induction of autophagy and apoptosis. Moreover, as shown in Fig. 7B, the levels of p53 were gradually increased and peaked at 6 h after MML treatment and then dramatically decreased. However, the phosphorylation levels of AMPK and ACC were increased at 9 h after MML treatment, whereas the level of phospho-mTOR was decreased. The level of LC3-II form was increased in a time-dependent manner which began at 6 h and then gradually increased, verifying autophagy induction by MML treatment. Subsequently, the cleaved forms of caspase-3 and PARP were detected at 9 h treatment with MML and were reached a maximal level at 24 h.

To clarify whether MML-triggered AMPK activation leads to autophagy induction, the protein levels related to AMPK signaling pathway were analyzed by immunoblotting after a 24-h treatment with compound C (comp C) known as AMPK inhibitor. As shown in Fig. 7C, pretreatment with comp C significantly decreased phospho-AMPK and phospho-ACC levels compared to MML treatment alone, whereas phosphorylated mTOR was increased. As a result, LC3 II level was clearly decreased by comp C, indicating autophagy suppression by AMPK inhibition. These results clearly suggest that MML-induced autophagy is activated by AMPK activation. To further confirm the relationship between p53 and AMPK in autophagy induction, we constructed p53 knockdown cell line and then levels of p53 and phosphorylated AMPK were analyzed by immunoblotting after MML treatment. As shown in Fig. 7D, p53 level was markedly decreased in MML-treated p53 knockdown cells (shp53) compared with MML-treated shControl cells. Moreover, phosphorylated levels of AMPK and ACC were also remarkably increased in MML-treated p53 knockdown cells, whereas phospho-mTOR level was decreased. Consequently, p53 silencing enhanced LC3 II level, which indicates autophagy induction. Taken together, these results suggest that MML treatment triggers autophagy via p53-mediated AMPK/mTOR signaling pathway, which leads to apoptosis in A549 cells.

On the other hand, recent studies revealed that MAP kinase signaling pathway, including ERK1/2 and p38, plays an important role in regulating autophagy induction [30], [37], [44], [45]. To investigate whether activation of MAP kinases by phosphorylation is involved in MML-induced autophagy, therefore, when the levels of the phosphorylated forms of ERK1/2 and p38 were examined by immunoblotting, any change in their levels by MML treatment was not observed (S2 Figure).

## Discussion

Several natural compounds derived from plants were recently reported to cause cancer cell death by induction of autophagy in various human cancer cells [6], [7], [18]–[20], [30]. In this study, we have shown for the first time that mimulone (MML), a compound from the *Paulownia tomentosa* fruits, induces autophagy as well as caspase-mediated apoptosis in human lung cancer cells. Furthermore, we firstly found that autophagy inhibition significantly attenuated apoptosis, which is in contrast with the previous results reported to date [7], [29], [35]–[40], [43]–[46]. Our results presented the MML could prevent cell proliferation and trigger caspase-mediated apoptotic cell death in A549 cells, as demonstrated by MTT assay, Annexin V/PI double staining, caspase inhibition and PARP cleavage following caspase-3 activation. MML treatment also triggered autophagy, as evidenced by MDC staining, LC3 immunostaining and the accumulations of LC3-II, Beclin-1 and ATG7, which are commonly known as methods for monitoring autophagy [33].

LC3-II, Beclin-1 and ATG7 play indispensable roles in autophagosomes formation and especially LC3-II is the most reliable biomarker of autophagy, as it is well established that LC3-II levels are directly proportional to autophagosome formation [33]–[39]. In this study, we demonstrated that MML augments LC3-II, Beclin-1 and ATG7 levels in a dose-dependent way in A549 cells, as characterized by immunoblotting. This result is in agreement with recent reports [19], [20] that paclitaxel and feroniellin A, natural compounds from plant, enhanced Beclin-1 and LC3-II levels in A549 cells. Moreover, our result demonstrated that MML triggers autophagy without interfering with autophagic flux in A549 cells, as evidenced by the changes of p62 and LC3-II levels after treatment of MML and CQ, which is consistent with a recent report [47] that docosahexaenoic acid (DHA) induced autophagy without impairment of autophagic vesicle turnover.

Many recent reports have demonstrated that autophagy inhibition by pharmacologic inhibitors, such as 3-MA and wortmannin, or genetic knockdown of several important autophagy-related genes, such as *ATG5*, *ATG7* and *Beclin-1*, enhance apoptosis induced by natural compounds or other chemotherapeutic agents, indicating that autophagy provides a protective mechanism against apoptosis induced by anticancer drugs [7], [30], [35]–[40], [43]–[46]. In addition, a recent study also reported that paclitaxel-mediated apoptotic cell death in A549 cells was promoted by pretreatment with 3-MA or knockdown of *Beclin-1* gene [19]. Contrary to these reports, however, our present study clearly demonstrated that autophagy inhibition by 3-MA and knockdown of *Beclin-1* gene significantly decreases MML-induced apoptosis in A549 cells, as indicated by decreased levels of morphological and biochemical characteristics, including cell viability, Annexin V/PI-positive cells, caspase-3 and PARP cleavages, LC3 puncta accumulation as well as MDC-positive vacuole formation and Beclin-1 and LC3-II protein levels. Our results are consistent with a recent report [20] that repression of Beclin-1 expression by siRNA decreased FERO-induced apoptosis in A549RT-eto cells. These results suggest that autophagy can serve as a cytotoxic mechanism against apoptotic cell death induced by natural compounds.

It is well documented that inhibition of p53 by a pharmacological inhibitor or gene knockdown induces autophagy [39]–[41]. In the present study, our result demonstrated that MML significantly reduces the protein level of p53 and consequently induces autophagy in A549 cells.

Autophagy process is known to be regulated by MAP kinase signaling pathway, including ERK1/2 and p38 [37], [45], and recent studies also proved that autophagy in some types of cancer cells was triggered through ERK1/2 activation by natural compounds, including dendropanoxide [30], aristolochic acid I [44], and daunorubicin [48]. In the current study, we found that MML-induced autophagy is not associated with activations of ERK1/2 and p38 pathways. Instead, our results clearly showed that MML triggers autophagy through activation of AMPK and inhibition of mTOR. Moreover, AMPK inhibition by comp C significantly decreased autophagy induction, which leads to reduced cytotoxic effect by MML, demonstrating MML-induced autophagy through activation of AMPK and inhibition of mTOR. On the other hand, autophagy induced by p53 loss is well known to be closely associated with activation of AMPK and inhibition of mTOR [39]–[43]. Similarly, the present results also revealed that shRNA-mediated depletion of p53 enhanced activation of AMPK and inhibition of mTOR, which resulted in a significant augment of autophagy induction in A549 cells. Taken together, our results demonstrated that the decrease in p53 levels by MML enhances induction of both autophagy and apoptosis through p53-mediated AMPK/mTOR signaling pathway in A549 cells with wild-type p53. These results are in accordance with a recent report [47] that docosahexaenoic acid (DHA) induced autophagy via p53/AMPK/mTOR signaling and enhanced apoptotic cell death in human cancer cells with wild-type p53. On the other hand, considering that DHA may induce autophagy via other mechanisms besides the p53 pathway [47], it is possible that MML-triggered autophagy may be also mediated in part by p53-independent mechanisms.

In conclusion, we have presented for the first time that MML treatment in human lung adenocarcinoma A549 cells induces autophagy via p53-mediated AMPK/mTOR signaling and autophagy inhibition reduces MML-induced apoptosis. Our data in this study suggest that MML may be potential new drug candidate for treating human lung adenocarcinoma. To verify whether MML can be a clinically useful anti-cancer agent for treatment of human lung adenocarcinoma, further studies are needed to assess the potential toxicity of MML to normal tissues and its ability to suppress tumor growth in a subcutaneous human lung cancer xenogarft model.

## Supporting Information

S1 Figure MML increases sub-G1 phase in A549 cells. (A) A549 cells were treated with MML at different concentrations (0–60 µM) for 24 h and stained with PI. Cell cycle was analyzed by flow cytometer. (B) Bar graph indicates the percentage of sub-G1 phase cells. Data were expressed as mean ± SEM of three independent experiments. *p<0.05 compared with control.
(TIF)

S2 Figure MML-induced autophagy was not involved in MAPK signaling pathway. (A) A549 cells were treated with 60 µM of MML for time-dependent manner and then immunoblot analysis was performed with antibodies against p-p38, p38, p-ERK 1/2 and ERK 1/2, respectively. GAPDH was used as loading control of immunoblotting. (B) Bar graph represents densitometry analysis of p38 and ERK. Data were expressed as mean ± SEM of three independent experiments. N.S: no significance.
(TIF)

## References

[1] Jemal A, Siegel R, Ward E, Hao Y, Xu J, et al (2009) Cancer statistics, 2009. CA Cancer J Clin 59:225–249. 19474385 10.3322/caac.20006

[2] Ramalingam SS, Owonikoko TK, Khuri FR (2011) Lung cancer: New biological insights and recent therapeutic advances. CA Cancer J Clin 61:91–112. 21303969 10.3322/caac.20102

[3] Reck M, Heigener DF, Mok T, Soria JC, Rabe KF (2013) Management of non-small-cell lung cancer: recent developments. Lancet 382:709–719. 23972814 10.1016/S0140-6736(13)61502-0

[4] Cragg GM, Newman DJ (2005) Plants as a source of anti-cancer agents. J Ethnopharmacol 100:72–79. 16009521 10.1016/j.jep.2005.05.011

[5] Mehta RG, Murillo G, Peng X (2010) Cancer chemoprevention by natural products: how far have we come? Pharm Res 27:950–961. 20238150 10.1007/s11095-010-0085-y

[6] Tan W, Lu J, Huang M, Li Y, Chen M, et al (2011) Anti-cancer natural products isolated from Chinese medicinal herbs. Chin Med 6:27. 21777476 10.1186/1749-8546-6-27 PMC3149025

[7] Zhang X, Chen LX, Ouyang L, Cheng Y, Liu B (2012) Plant natural compounds: targeting pathways of autophagy as anti-cancer therapeutic agents. Cell Prolif 45:466–476. 22765290 10.1111/j.1365-2184.2012.00833.x PMC6496896

[8] Gomathinayagam R, Sowmyalakshmi S, Mardhatillah F, Kumar R, Akbarsha MA, et al (2008) Anticancer mechanism of plumbagin, a natural compound, on non-small cell lung cancer cells. Anticancer Res 28:785–792. 18507021

[9] Hsu HF, Houng JY, Kuo CF, Tsao N, Wu YC (2008) Glossogin, a novel phenylpropanoid from *Glossogyne tenuifolia*, induced apoptosis in A549 lung cancer cells. Food Chem Toxicol 46:3785–3791. 18976690 10.1016/j.fct.2008.09.068

[10] Wu SH, Hang LW, Yang JS, Chen HY, Lin HY, et al (2010) Curcumin induces apoptosis in human non-small cell lung cancer NCI-H460 cells through ER stress and caspase cascade- and mitochondria-dependent pathways. Anticancer Res 30:2125–2133. 20651361

[11] Mou H, Zheng Y, Zhao P, Bao H, Fang W, et al (2011) Celastrol induces apoptosis in non-small-cell lung cancer A549 cells through activation of mitochondria- and Fas/FasL-mediated pathways. Toxicol in Vitro 25:1027–1032. 21466843 10.1016/j.tiv.2011.03.023

[12] Chang WA, Lin ES, Tsai MJ, Huang MS, Kuo PL (2014) Isolinderalactone inhibits proliferation of A549 human non-small cell lung cancer cells by arresting the cell cycle at the G0/G1 phase and inducing a Fas receptor and soluble Fas ligand-mediated apoptotic pathway. Mol Med Rep 9:1653–1659. 24604009 10.3892/mmr.2014.2015

[13] Huang RY, Chu YL, Jiang ZB, Chen XM, Zhang X, et al (2014) Glycyrrhizin suppresses lung adenocarcinoma cell growth through inhibition of thromboxane synthase. Cell Physiol Biochem 33:375–388. 24556579 10.1159/000356677

[14] Zhang Y, Zhuang Z, Meng Q, Jiao Y, Xu J, et al (2014) Polydatin inhibits growth of lung cancer cells by inducing apoptosis and causing cell cycle arrest. Oncol Lett 7:295–301. 24348867 10.3892/ol.2013.1696 PMC3861602

[15] Warin RF, Chen H, Soroka DN, Zhu Y, Sang S (2014) Induction of lung cancer cell apoptosis through a p53 pathway by [6]-shogaol and its cysteine-conjugated metabolite M2. J Agric Food Chem. 62(6):1352–1362. 24446736 10.1021/jf405573e PMC3983336

[16] Song J, Ko HS, Sohn EJ, Kim B, Kim JH, et al (2014) Inhibition of protein kinase C α/βII and activation of c-Jun NH_2_-terminal kinase mediate glycyrrhetinic acid induced apoptosis in non-small cell lung cancer NCI-H460 cells. Bioorg Med Chem Lett 24:1188–1191. 24461294 10.1016/j.bmcl.2013.12.111

[17] Avisetti DR, Babu KS, Kalivendi SV (2014) Activation of p38/JNK pathway is responsible for embelin induced apoptosis in lung cancer cells: transitional role of reactive oxygen species. PLoS One 9(1):e87050. 24466324 10.1371/journal.pone.0087050 PMC3899364

[18] Hung JY, Hsu YL, Li CT, Ko YC, Ni WC, et al (2009) 6-Shogaol, an active constituent of dietary ginger, induces autophagy by inhibiting the AKT/mTOR pathway in human non-small cell lung cancer A549 cells. J Agric Food Chem 57:9809–9816. 19799425 10.1021/jf902315e

[19] Xi G, Hu X, Wu B, Jiang H, Young CY, et al (2011) Autophagy inhibition promotes paclitaxel-induced apoptosis in cancer cells. Cancer Lett 307:141–148. 21511395 10.1016/j.canlet.2011.03.026

[20] Kaewpiboon C, Surapinit S, Malilas W, Moon J, Phuwapraisirisan P, et al (2014) Feroniellin A-induced autophagy causes apoptosis in multidrug-resistant human A549 lung cancer cells. Int J Oncol 44:1233–1242. 24535083 10.3892/ijo.2014.2297

[21] Asai T, Hara N, Kobayashi S, Kohshima S, Fujimoto Y (2008) Geranylated flavanones from the secretion on the surface of the immature fruits of *Paulownia tomentosa* . Phytochemistry 69:1234–1241. 18206191 10.1016/j.phytochem.2007.11.011

[22] Schneiderová K, Slapetová T, Hrabal R, Dvořáková H, Procházková P, et al (2013) Tomentomimulol and mimulone B: two new C-geranylated flavonoids from *Paulownia tomentosa* fruits. Nat Prod Res 27:613–618. 22548348 10.1080/14786419.2012.683002

[23] Šmejkal K, Grycová L, Marek R, Lemière F, Jankovská D, et al (2007) *C*-geranyl compounds from *Paulownia tomentosa* fruits. J Nat Prod 70:1244–1248. 17625893 10.1021/np070063w

[24] Šmejkal K, Holubova P, Zima A, Muselik J, Dvorska M (2007) Antiradical activity of *Paulownia tomentosa* (Scrophulariaceae) extracts. Molecules 12:1210–1219. 17876290 10.3390/12061210 PMC6149512

[25] Kim SK, Cho SB, Moon HI (2010) Neuroprotective effects of a sesquiterpene lactone and flavanones from *Paulownia tomentosa s*teud. against glutamate-induced neurotoxicity in primary cultured rat cortical cells. Phytother Res 24:1898–1900. 20683844 10.1002/ptr.3277

[26] Navrátilová A, Schneiderová K, Veselá D, Hanáková Z, Fontana A, et al (2013) Minor *C*-geranylated flavanones from *Paulownia tomentosa* fruits with MRSA antibacterial activity. Phytochemistry 89:104–113. 23453910 10.1016/j.phytochem.2013.01.002

[27] Šmejkal K, Babula P, Slapetová T, Brognara E, Dall′Acqua S, et al (2008) Cytotoxic activity of *C*-geranylcompounds from *Paulownia tomentosa* fruits. Planta Med 74:1488–1491. 18729043 10.1055/s-2008-1081339

[28] Šmejkal K, Svačinova J, Šlapetova T, Schneiderova K, Dall′Acqua S, et al (2010) Cytotoxic activities of several geranyl-substituted flavanones. J Nat Prod 73:568–572. 20192247 10.1021/np900681y

[29] Kollár P, Bárta T, Závalová V, Šmejkal K, Hampl A (2011) Geranylated flavanone tomentodiplacone inhibits proliferation of human monocytic leukaemia (THP-1) cells. Br J Pharmacol 162:1534–1541. 21175584 10.1111/j.1476-5381.2010.01171.x PMC3057291

[30] Lee JW, Kim KS, An HK, Kim CH, Moon HI, et al (2013) Dendropanoxide induces autophagy through ERK1/2 activation in MG-63 human osteosarcoma cells and autophagy inhibition enhances dendropanoxide-induced apoptosis. PLoS One 8:e83611. 24358301 10.1371/journal.pone.0083611 PMC3866153

[31] Elmore S (2007) Apoptosis: a review of programmed cell death. Toxicol Pathol 35:495–516. 17562483 10.1080/01926230701320337 PMC2117903

[32] Boatright KM, Salvesen GS (2003) Mechanisms of caspase activation. Curr Opin Cell Biol 15:725–731. 14644197 10.1016/j.ceb.2003.10.009

[33] Mizushima N (2004) Methods for monitoring autophagy. Int J Biochem Cell Biol 36:2491–2502. 15325587 10.1016/j.biocel.2004.02.005

[34] Zhang X, Chen S, Huang K, Le W (2013) Why should autophagic flux be assessed? Acta Pharmacol Sin 34:595–599. 23474710 10.1038/aps.2012.184 PMC4002868

[35] Thorburn A (2008) Apoptosis and autophagy: Regulatory connections between two supposedly different processes. Apoptosis 13:1–9. 17990121 10.1007/s10495-007-0154-9 PMC2601595

[36] Notte A, Leclere L, Michiels C (2011) Autophagy as a mediator of chemotherapy-induced cell death in cancer. Biochem Pharmacol 82:427–434. 21704023 10.1016/j.bcp.2011.06.015

[37] Aredia F, Giansanti V, Scovassi AI (2012) Autophagy and cancer. Cell 1:520–534. 10.3390/cells1030520 PMC3901115 24710488

[38] Gump JM, Thorburn A (2011) Autophagy and apoptosis: what is the connection? Trends cell Biol 21:387–392. 21561772 10.1016/j.tcb.2011.03.007 PMC3539742

[39] Levine B, Abrams J (2008) p53: The Janus of autophagy? Nat Cell Biol 10:637–639. 18521069 10.1038/ncb0608-637 PMC2739720

[40] Tasdemir E, Maiuri MC, Galluzzi L, Vitale I, Djavaheri-Mergny M, et al (2008) Regulation of autophagy by cytoplasmic p53. Nat Cell Biol 10:676–687. 18454141 10.1038/ncb1730 PMC2676564

[41] Chiara Maiuri M, Galluzzi L, Morselli E, Kepp O, Ahmad Malik S, et al (2010) Autophagy regulation by p53. Curr Opin Cell Biol 22:181–185. 20044243 10.1016/j.ceb.2009.12.001

[42] Kim J, Kundu M, Viollet B, Guan KL (2011) AMPK and mTOR regulate autophagy through direct phosphorylation of Ulk1. Nat Cell Biol 13:132–141. 21258367 10.1038/ncb2152 PMC3987946

[43] Mihaylova MM, Shaw RJ (2011) The AMPK signalling pathway coordinates cell growth, autophagy and metabolism. Nat Cell Biol 13:1016–1023. 21892142 10.1038/ncb2329 PMC3249400

[44] Zeng Y, Yang X, Wang J, Fan J, Kong Q, et al (2012) Aristolochic acid I induced autophagy extenuates cell apoptosis via ERK 1/2 pathway in renal tubular epithelial cells. PLoS One 7:e30312. 22276178 10.1371/journal.pone.0030312 PMC3262826

[45] Cagnol S, Chambard JC (2010) ERK and cell death: Mechanisms of ERK-induced cell death apoptosis, autophagy and senescence. FEBS J 277:2–21. 19843174 10.1111/j.1742-4658.2009.07366.x

[46] Livesey KM, Tang D, Zeh HJ, Lotze MT (2009) Autophagy inhibition in combination cancer treatment. Curr Opin Investig Drugs 10:1269–1279. 19943199

[47] Jing K, Song KS, Shin S, Kim N, Jeong S, et al (2011) Docosahexaenoic acid induces autophagy through p53/AMPK/mTOR signaling and promotes apoptosis in human cancer cells harboring wild-type p53. Autophagy 7:1348–1358. 21811093 10.4161/auto.7.11.16658 PMC3242799

[48] Han W, Sun J, Feng L, Wang KF, Li D, et al (2011) Autophagy inhibition enhances daunorubicin-induced apoptosis in K562 Cells. PLoS ONE 6:e28491. 22164300 10.1371/journal.pone.0028491 PMC3229606

