# Supplementary figures and images for: Mimulone-Induced Autophagy through p53-Mediated AMPK/mTOR Pathway Increases Caspase-Mediated Apoptotic Cell Death in A549 Human Lung Cancer Cells
Source: PLoS One. 2014 Dec 9;9(12):e114607. doi: 10.1371/journal.pone.0114607 (PMC4260910; doi:10.1371/journal.pone.0114607)

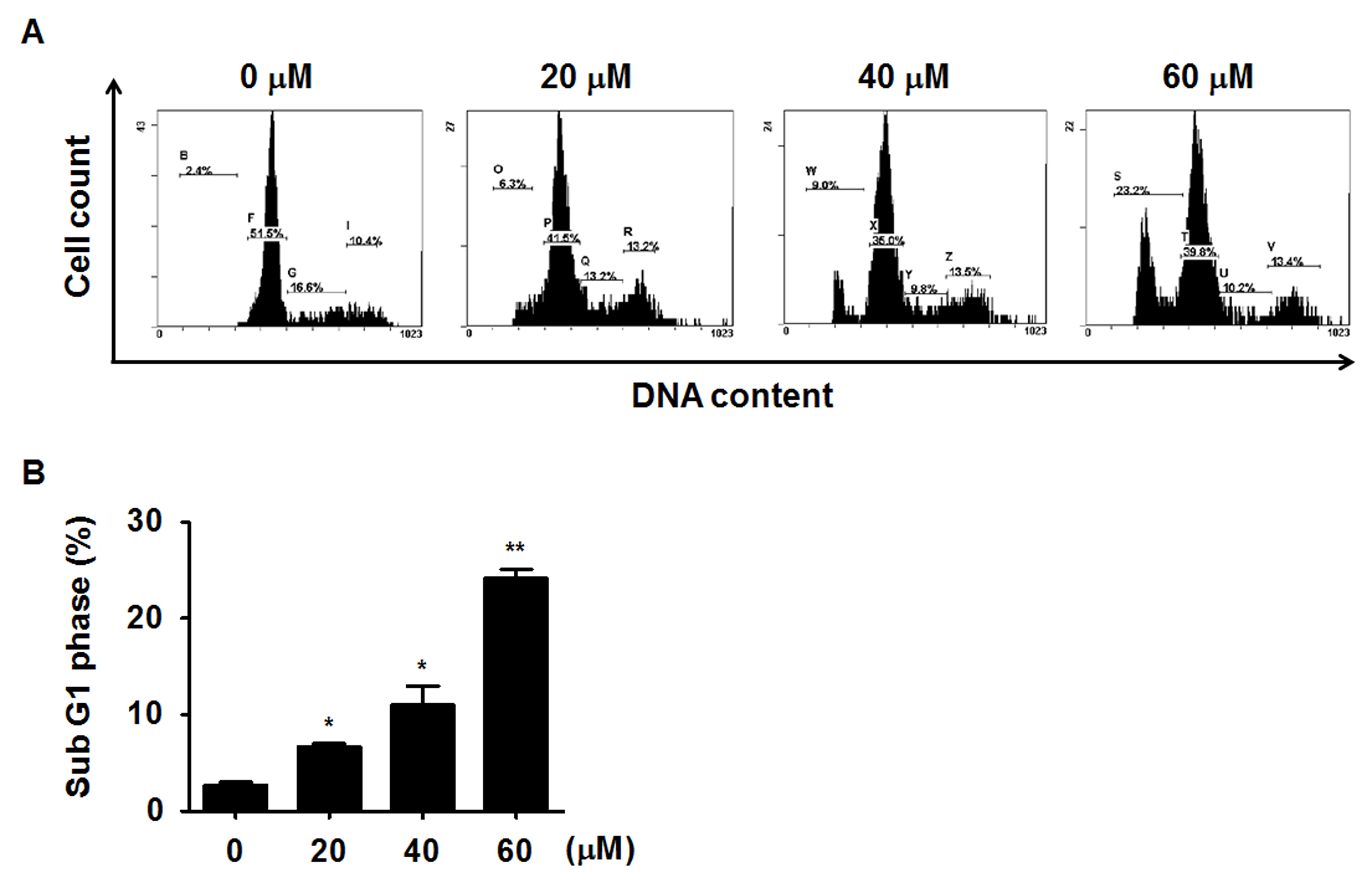

Supplement: S1 Figure — MML increases sub-G1 phase in A549 cells. (A) A549 cells were treated with MML at different concentrations (0–60 µM) for 24 h and stained with PI. Cell cycle was analyzed by flow cytometer. (B) Bar graph indicates the percentage of sub-G1 phase cells. Data were expressed as mean ± SEM of three independent experiments. *p<0.05 compared with control. (TIF) [file pone.0114607.s001.tif]

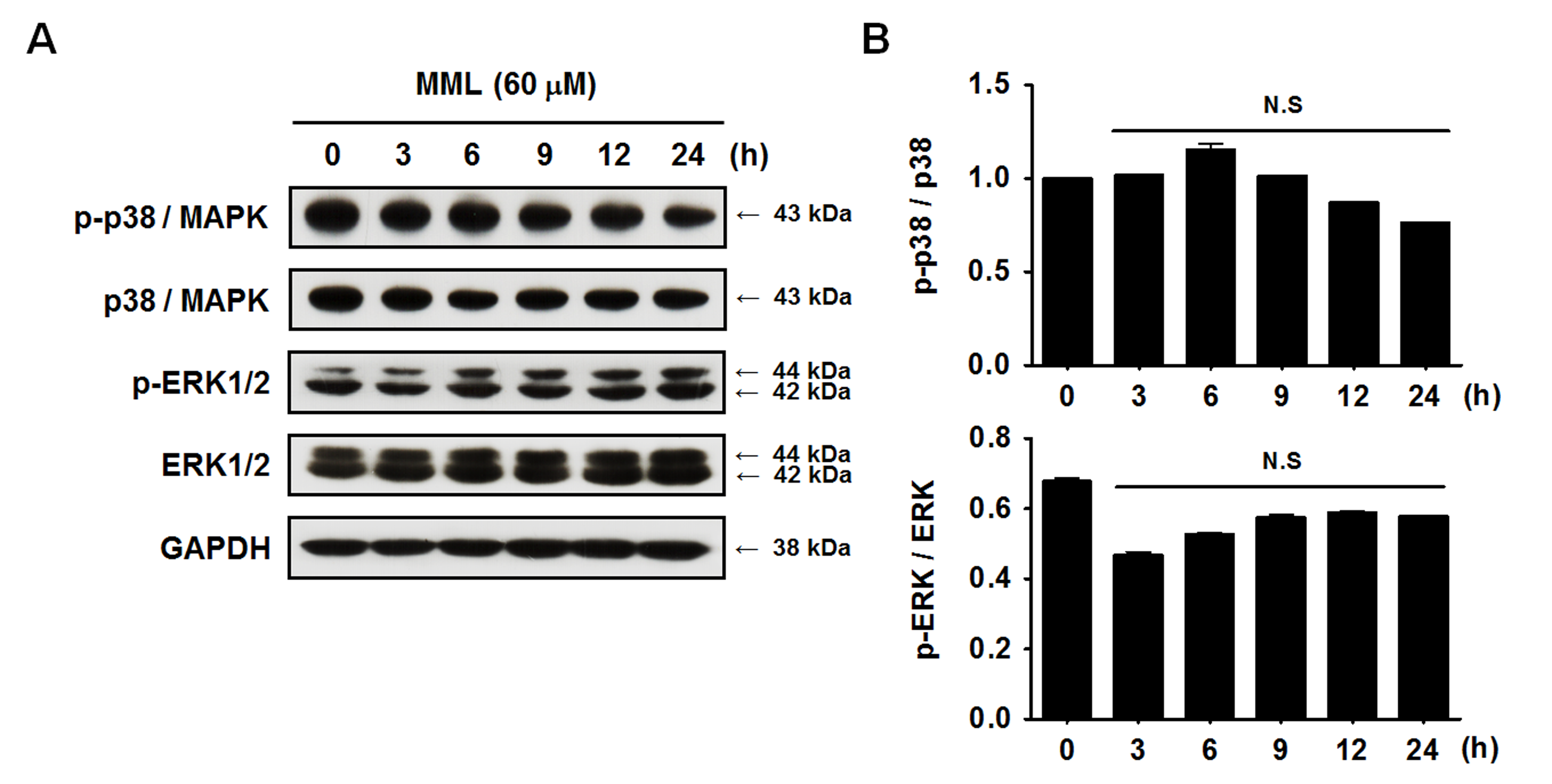

Supplement: S2 Figure — MML-induced autophagy was not involved in MAPK signaling pathway. (A) A549 cells were treated with 60 µM of MML for time-dependent manner and then immunoblot analysis was performed with antibodies against p-p38, p38, p-ERK 1/2 and ERK 1/2, respectively. GAPDH was used as loading control of immunoblotting. (B) Bar graph represents densitometry analysis of p38 and ERK. Data were expressed as mean ± SEM of three independent experiments. N.S: no significance. (TIF) [file pone.0114607.s002.tif]
